# Hospital and Institutional News

**Published:** 1914-12-19

**Authors:** 


### HOSPITAL AND INSTITUTIONAL NEWS.

### OUR CHRISTMAS APPEAL NUMBER.

We publish this week, as usual, a Special Christmas Appeal Number, which appears as an extra Supplement, and which sets forth the needs of the hospitals, proving, in fact, a guide to all who wish to know what are the requirements of the various, mainly London, institutions. This year, naturally, while the voluntary hospitals' needs are greater than ever, they have a unique opportunity for claiming the generous support of the nation, from the fact that they are doing double work, and taking on their shoulders in addition to the normal demands upon them a very important share in tending the nation's sick and wounded soldiers. What this means to the hospitals and to laymen alike is pointedly illustrated in the special articles which accompany the concisely drafted paragraphs. Apart from an illuminating article on the Financial Outlook, which deals succinctly and forcibly with the complex problems of hospital finance at the present crisis, there are two striking articles on "Soldiers as Apostles of the Voluntary System" and "The Christmas Spirit in War Time," which cannot fail to appeal alike to the lay and the institutional reader. We trust that every effort on the part of our readers will be made to circulate this Appeal Number, since the wider is its distribution the better will be the understanding by the general public of the degree to which they are indebted to the voluntary hospitals at the present time.

### OUR WAR CORRESPONDENTS AND CHRISTMAS WEEK.

We would draw the attention of our Special Correspondents who are providing us with Hospital War News each week from the different centres, large and small, that, owing to Christmas, we are compelled in our next issue, dated December 26, to go to press a day earlier than usual. Will they, therefore, take note and kindly oblige us by sending their special contributions in time to arrive not later than *the first post on Monday morning next*, December 21? The ready co-operation so far shown by our Correspondents in the different institutions assures us that in the few days elapsing from the time that the present Number of The Hospital is in their hands and the above date they will do their best, and make a special effort to assure that the customary complement of War News shall be received at our office in time to be incorporated in our next issue. Christmas this year cannot but be associated with War News, and therefore readers and writers will alike feel that the Number of The Hospital dated December 26 should contain an account of current news concerning the hospitals and their treatment of the wounded. To secure this, both in our next issue and in the following Number—the first of 1915—may, and we fear must, demand a little extra trouble and forethought. But the spirit of co-operation has been so pleasant and successful up to date that we feel no doubt that during the Christmas period extra efforts will be made to secure no falling off in this department.

### THE POOR-LAW INFIRMARY ON THE EVE OF CHANGE.

At the last meeting of the Leicester Guardians a report was presented of an interview between a deputation from the Guardians and Dr. Fuller, the Medical Inspector for Poor-Law purposes of the Local Government Board. If the statements in the report attributed to Dr. Fuller correctly describe what he said, the Poor-Law Infirmary would appear to be on the eve of revolutionary changes. The Leicester Guardians had suggested a staff of one nurse to ten beds. Dr. Fuller is reported to have said not only that this was totally inadequate, but that his own previous suggestion of one nurse to eight patients might be considered obsolete. In the future he should raise no objection to a scale based upon that of the voluntary hospitals, namely, two nurses to seven patients. He hoped the Guardians would ask at once for a staff of one nurse to six patients in the adult wards and of one to four in the children's wards. He pointed out that the proposed operating theatre was an advance upon the Local Government Board model plans, but to this he did not object, as they were prepared for advancement. Advances of a most important and far-reaching character were going to be made in the near future in all modern Poor-Law infirmaries. It is something inspiringly novel to find an official of the Local Government Board expressing views on staffing of such an advanced character. If the deputation has made no mistake, the visions of our Poor-Law reformers of a really efficient system of Poor-Law medical relief may not be so very far from realisation as some of them think.

### THE VOLUNTARY SPIRIT IN HOSPITALS AND ARMIES.

Apart from the natural exaggerations of patriotism, which body of men, our recruits or German conscripts, have given the best account of themselves in this war? There is no doubt about the answer: for when numbers have been pitted against enthusiasm and the doggedness of voluntary effort, then, unless the odds were absolutely overwhelming, the voluntarily enlisted body has proved the one more likely to win. It is the old question of quality *versus* quantity, courage and determination against numbers and big guns, and, on the whole, quality has it. Even our friends the French are not relying on their lines alone; it is the appeal of an invaded country against an old enemy which, in spite of the pacificists, has united her soldiers, among whom, it is safe to say, desertions have been fewer than among the Germans, whose system on occasion seems to have led them to train machine guns on their men rather than to trust to them. Under such circumstances what sane man would not desert? Yet the above are the respective fruits of compulsory militant conscription and voluntary service, and it is an encouraging contrast for all workers in the voluntary hospitals to observe.

### THE INSURANCE ACT AND HOSPITAL BEDS.

Some interesting speeches were made at the recent annual meeting of the Victoria Infirmary, Glasgow, with reference to the Insurance Act, the waiting list, and hospital beds. Mr. William Gray remarked concerning the proposed extension of the hospital, which they had decided to postpone until the war was over, that it was necessary, owing to the fact that the number of those waiting admission was equal to the whole accommodation of the institution. It was satisfactory to note that the general subscriptions had been practically the same as last year, and that employees' contributions showed an increase of £134, notwithstanding the Insurance Act. Colonel Roxburgh said that there was no doubt that admissions to all hospitals were increasing. The Western Infirmary, with its 600 or so beds, had between 700 and 800 waiting, and the proportion at the Victoria was very much the same. If the best results were to be obtained from the Insurance Act, there was no doubt that it would be necessary to increase the hospital accommodation. They would be very sorry to see the voluntary principle in any way interfered with. It is well, we think, to note this tendency, the force of which is now apt to be in a sense discounted, owing to our preoccupation with the war. But if the voluntary principle is to be preserved, and at the same time, if the question of increased accommodation is to be dealt with, there is no other way than that which we have outlined so often in The Hospital—namely, for a great co-operative scheme, under which Government loans should be given for building proposals, as noted once more in The Hospital Special Christmas Appeal Number this week.

### HOSPITALS AND PANEL DOCTORS.

A Special Committee of the London Insurance Committee, which has had under consideration the arrangements for the provision of medical benefit in the various metropolitan boroughs, pays a handsome and well-deserved tribute to the work of the voluntary hospitals in this connection. They say that throughout their investigation they have given very careful consideration to the arrangements obtaining with regard to the provision of specialist treatment for the insured persons at the various hospitals in the areas which have been under consideration, and they have noted with great satisfaction the cordial co-operation which exists between the practitioners on the panel and the staffs of the great hospitals in the district. They understand that consultative help is obtained from the staffs of the hospitals in such cases without any payment being required. They also consider that many of the difficulties in the medical service are of such a character as can be overcome by still greater co-operation of hospital authorities and general practitioners. The committee gathered from the hospital representatives who gave evidence that the out-patient departments of the hospitals have, since the introduction of the medical benefit, been relieved of the treatment to a considerable extent of trivial and chronic patients, and this has, of course, increased the number of patients treated by practitioners on the panel. On the other hand, they have been informed that there is not sufficient in-patient accommodation at the hospitals, and this lack of accommodation is now accentuated owing to the war. The need for additional accommodation is said to be particularly noticeable in the case of women patients. On this last statement, we venture to think that opinion is undecided. What, however, is only too certain is that the operation of the National Insurance Act has so much increased the demands on hospital beds as to make further provision in this direction an absolute and pressing necessity, as a reference to our Note on "The Insurance Act and Hospital Beds" is sufficient to show.

### A DOCTOR DISPATCH-RIDER.

Among those who have lately joined the corps of motor-cycle dispatch-riders for active service is Dr. Alexander Beck Cluckie, of Gay Street, Bath, who is an M.B., Ch.B., and one of the honorary surgeons to the local eye infirmary. Dr. Beck Cluckie previously held the appointments of assistant ophthalmic surgeon to the Greenock Eye Infirmary and senior house surgeon at the Royal London Ophthalmic Hospital, City Road. He is also a keen Rugby footballer.

### CAN A PANEL DOCTOR'S FEES BE ATTACHED?

An interesting case, concerning the right to garnishee sums payable to a panel doctor in respect of a judgment debt, has been decided by Mr. Justice Rowlatt in the King's Bench Division. The plaintiffs were the executors under the will of Dr. John O'Leary O'Driscoll, a Manchester medical man, who died in 1910, and the defendants were the Manchester Insurance Committee. Counsel for the plaintiffs declared that the case was the result of certain questions raised on a motion to make absolute a conditional order of garnishee which the plaintiffs obtained. An issue was directed as to what sums, if any, were due to the defendants in the action by the garnishee. The late doctor's only assets were the goodwill of his practice, which the executors had sold for £380 to a Dr. Sweeney, who paid part on account and a writ was issued against him for the rest. Last March judgment was recovered against him for £246 odd and £9 costs, and that was the judgment debt in respect of which the subsequent application for attachment was made. It was useless, said counsel, to issue execution against Dr. Sweeney, as he had no assets, the furniture being settled on his wife. In April the plaintiffs obtained an order of attachment of moneys due or accruing to Dr. Sweeney in respect of his duties as a panel doctor in Manchester. The order was served on the Committee, who contended that it was contrary to public policy that money payable for public services under the Act should be attachable; and, further, that under the Act and under their arrangements with Dr. Sweeney and other panel doctors in Manchester, there was no debt due which under the jurisdiction of the Court in the matter of the garnishee could be made the subject-matter of attachment. The plaintiffs argued that the point about public policy was untenable, because the Insurance Committee under Section 30 of the Act were constituted a corporate body and might sue or be sued. The Committee were debtors for the amounts due to the Manchester doctors, and, therefore, counsel contended that the amounts due to Dr. Sweeney could be attached. The Judge held that there was a debt accruing to Dr. Sweeney from the Committee in respect of the year 1913, and in his opinion the fees of panel doctors could be attached. The plaintiffs were entitled to an order attaching amounts due to Dr. Sweeney. Leave to appeal was granted.

### ST. KATHARINE'S HOSPITAL REFORM SCHEME.

As announced in The Hospital at the time it was drafted, the new scheme for restoring St. Katharine's Hospital and its work to the East End of London, which was decided on early in the year, determined to establish a college for the training of health visitors. A practical step in the direction of carrying out the scheme which, on the request of Queen Alexandra, was drawn up by the Lord Chancellor and his advisers, is that a house has been taken at Poplar where the proposed health visitors may be trained. It will be recalled by our readers that the Regent's Park establishment is gradually being superseded, and that the women who are to be trained as health visitors will work among the poor under a scheme which places their supervision in the hands of the local medical officers of health.

### THE NEW CHAIRMAN OF LEICESTER ROYAL INFIRMARY.

It is fitting that Sir Edward Wood should be succeeded in the chairmanship of the board by a friend with whom he has long been associated in business enterprise. In Mr. H. Simpson Gee, J.P., the institution will be guided by a gentleman whose reputation as a financier and successful business-man requires no commendation. Mr. Simpson Gee has on many occasions rendered valuable help to the institution and contributed to its various funds. He is well known in the banking world as one of the directors of the London City and Midland Bank, and locally as chairman of the directors of Stead and Simpson, Ltd., one of the multiple trading firms, as deputy-chairman of the directors of Freeman, Hardy and Willis, Ltd., chairman of R. Hornsby and Sons, Ltd. (Grantham), the Bagworth (Leicester) Colliery Company, and other local enterprises. His election to the chairmanship was moved by Sir William Vincent, J.P., and seconded by Sir Arthur Hazlerigg, Bart. No doubt can be suggested that in these important administrative changes Leicester Royal Infirmary will continue to maintain its established efficiency.

### A SUGGESTED HOME FOR DOMESTIC SERVANTS.

There is still a good deal to be done, as Sir Edward Wood outlined in his letter of resignation. The reconstruction of the only remaining wing of the old buildings, containing the accident and St. Luke's wards, should be kept well to the front in the institution's programme, as well as the suggested Home for domestic servants. It has been practically decided to proceed at once with the erection of a modern pathological department and the reconstruction of the mortuary, the need for which was emphasised by Sir Henry Burdett on his recent visit to the institution. The financial position of the institution will also be closely observed and consolidated by the new chairman, whose advice in this connection will be of the utmost gain. In conclusion we may add that we have frequently prophesied in these columns that domestic Homes for the servants in hospitals would one day become the rule, since the attention given in the past to improving the nurses' quarters has reacted beneficially already on that accorded to the maids.

### AN ISOLATION HOSPITAL FOR DUBLIN.

The Public Health Committee of Dublin has long had pressed upon its consideration the desirability of providing an isolation hospital in the city, and matters have now reached the point of a definite proposal. This is that an isolation hospital should be built on the ground which lies between the Tuberculosis Hospital and the Coastguard Station on the Pigeon-house Road. Perhaps the *prima facie* recommendation of such a site is that its proximity to the Tuberculosis Hospital would prevent the outcry against its existence which is invariably raised whenever there is a suggestion that a sanatorium or an infectious diseases hospital should be built. We have dealt with the absurdity of this dread, in either case, both from the medical and the commercial point of view too often again to insist upon it in the present case, and can here merely call attention to the grounds which have recently been put forward in support of the contention that an isolation hospital is essential to the city. Chief among the protagonists on this side of the question is the Medical Inspector of the Local Government Board, Dr. Browne, who calls attention to the existing risks from an outbreak of smallpox, plague, or cholera. Sir Charles Cameron is also quoted as remarking that among the urgent reasons why a moderate-sized isolation hospital should be provided at an early date is the fact that typhoid fever and Asiatic cholera have, he says, appeared among the Continental armies. Prisoners from them, he adds, putting thus all the risk on our enemies, might act as carriers, and on arriving in Ireland spread the infection. Whether this aspect of the case, and the prevalent war fever, will succeed in doing what the Medical Inspector of the Local Government Board and other competent authorities have failed to do, remains to be seen; but at all events a topical argument has been found, and there is much virtue in the tactics implied by it.

### REFUSAL TO INCREASE A MEDICAL SUPERINTENDENT'S SALARY.

The Local Government Board have notified the Camberwell Board of Guardians that, having given the matter their careful consideration, in view of all the circumstances, they are not at present prepared to sanction an increase of £50 a year in the salary of Dr. Keats, medical superintendent of the infirmary. The Guardians in the early part of last year decided to increase Dr. Keats' salary by £100 a year, but the Local Government Board at the time would consent only to an increase of £50. The present refusal to grant the further £50 has not been accepted with resignation by the Guardians, who have referred the matter to the Finance Committee to see if something cannot be done to induce the authorities at Whitehall to give way.

### GENERAL POST OF MEDICAL SUPERINTENDENTS.

The Metropolitan Asylums Board lately agreed to the recommendation of the Hospitals Committee that Dr. Rickett be transferred from his present post of medical superintendent of the River Hospitals and Ambulance Service to the post of medical superintendent of the Park Hospital, subject to the assent of the Local Government Board. If the Local Government Board agree to that arrangement the Managers of the Asylums Board propose to transfer Dr. Cameron from the Downs Sanatorium to succeed Dr. Rickett as medical superintendent of the River Hospitals and Ambulance Service. The impending vacancy at the Park Hospital is consequent upon Dr. Birdwood having given notice of resignation. The admission of infectious patients to the Park Hospital was commenced on November 2, as soon as the ringworm cases were removed.

### A SCOTTISH TRAINING SCHOOL FOR INSTITUTION OFFICIALS.

Mr. James A. J. Currie, house steward of the recently opened Colony for Epileptics at Chryston, Glasgow, has been appointed house steward of The Retreat (Mental Hospital), York. Mr. Currie received his institutional training at the Glasgow District Mental Hospital, Lenzie, the largest of its kind in Scotland, under Mr. James S. Marr, the well-known house steward of that hospital. Within recent years a number of Mr. Marr's assistants have been similarly promoted: Mr. David F. Peters having been appointed house steward of the Aberdeen City Mental Hospital at Kingseat; Mr. John Bigley house steward of the Renfrew District Mental Hospital at Dykebar, Paisley; Mr. William Hill house superintendent at the Glasgow Royal Maternity and Women's Hospital; and now Mr. Currie has been promoted to York. From this it can be understood that the Glasgow District Mental Hospital is regarded as an excellent training school, and no small amount of credit is due to Mr. Marr for turning out so many men whose promotion bears evidence to the value of their training.

### A DANGEROUS UNQUALIFIED DOCTOR.

A remarkable career of illegality was revealed last week, when John Cubbin was charged before the Recorder with giving false death certificates and with committing perjury. The prosecution stated that the death certificates were signed by the prisoner in the name of Harrison, describing himself as "M.R.C.S., L.R.C.P." In 1900 the prisoner was convicted of giving a false medical certificate, and subsequently had various terms of imprisonment imposed on him, including five years' penal servitude for bigamy in 1903 and five years for a similar offence in 1908. In the name of "Dr. Monroe" the prisoner, who had had some medical training, became assistant to a London doctor, but he left that employment last January and representing himself to Dr. Allen, who had a dispensary in Bethnal Green, as Surgeon-Major Harrison, offered his services and was engaged as an assistant by Dr. Allen, who believed him to be a medical man. Later the prisoner, who pretended that he had inherited a baronetcy, printed this title and other distinctions on his visiting cards. It was only fair, added counsel, to say that he had treated his patients with skill. In defence the prisoner stated that he had received medical training at Montreal and Vienna, and that there was some good in him ready to "burst into flame" if he were given another chance. Dr. Allen, in reply to the Recorder, stated that there were no complaints about him, and that patients spoke well of the prisoner. The Recorder, in sentencing him to three years' penal servitude, remarked that he was a very dangerous man.

### MEDICAL TREATMENT AND POOR-LAW DISQUALIFICATION.

The Charity Commissioners have recently forwarded to the London County Council a draft amending the scheme under which the non-ecclesiastical charities of Streatham are administered. It is suggested that provision shall be made empowering the trustees to remove any almsperson or pensioner who receives Poor-Law relief other than medical relief; or is detained as a person suffering from mental disease; or becomes an inmate of any Poor-Law institution for the purpose of receiving medical relief. The new provisions are similar to those which have been introduced into many new schemes prepared by the Charity Commissioners, and the only point arising is that the clause will operate harshly on pensioners who become disqualified owing to their entering an infirmary to receive surgical or medical treatment. In other cases the Council have suggested to the Commissioners that the disqualification arising from becoming the inmate of a Poor-Law institution for the purpose of receiving medical relief should not operate until after a residence of four weeks in the institution. This point is still under the consideration of the Commissioners, and it is proposed to repeat the suggestion in connection with the present scheme.

### HOSPITAL CHEER AND A QUIET CHRISTMAS.

In spite of the fact that the voluntary hospitals depend upon an intelligent public interest in everything which concerns them, and on the close intercourse between the institution and the world in general, it is remarkable how few, comparatively speaking, in our immense population other, of course, than actual hospital patients have ever visited a hospital ward, much more spent Christmas there. Yet everyone is welcome to do so. Hospitals, for no canonical reasons, do as a rule keep Christmas practically over the octave, or, in other words, generally make a week of it, and this year the spirit will be the same, though the festivities will be fewer. In children's hospitals everything will go on as far as possible as usual: the ward decorations, the coloured lights, the crackers, the concert in the out-patient department, and the Christmas-tree. But in general hospitals, for grown-ups, the Christmas procession of Santa Claus, the clowns, the concert in each ward, will probably be abandoned, as the reduced staff have no time for such things; but the Christmas fare, the presents to each patient, and ward decorations will be, generally speaking, the same. It is not the patients or those who tend them that will be joyless, but rather the strong and the well outside.

### HOSPITAL POEMS.

Poetry inspired by hospitals is, perhaps fortunately, rare, and divides itself broadly into two classes. The first, as every person interested in literature is aware, comprises Henley's well-known verses, realistic in aim and technically good; the second is read as a rule only by hospital staffs, unless it finds its way into a local newspaper, and consists of a patient's appreciation in verse of the treatment which he has received. Examples of this latter class arrive periodically at The Hospital office, and are carefully read by the Editor in the faint but inextinguishable hope of finding them good enough for publication. The current number of the *Author*, however, contains the following announcement: "'Dies Iræ and Angelus' is the title given by Miss Meta Orred to a small collection of poems which she is publishing for the benefit of the base hospitals at the price of 6d. net. We commend these poems by the author of the words of 'In the Gloaming,' and hope the hospitals will benefit by their appearance." In that hope we share, although up to the present we have had no opportunity of reviewing them.

### PANEL PRACTITIONERS AND TUBERCULOUS PATIENTS.

At a meeting of the Middlesex Insurance Committee last week the report of the Sanatoria Committee was considered. This report recommended that the National Insurance Commissioners be asked to alter the present method of remuneration to doctors for attendance on patients receiving domiciliary treatment. In the view of the Committee this treatment, instead of having a special fee, should be included under one capitation fee, to include all ailments, payments being made out of the medical benefit fund. Dr. Brackenbury opposed on the grounds that this proposal was an attempt to cut down the incomes of Panel practitioners. The Chairman said that it was desirable that all services rendered by medical practitioners should be paid for out of one fund, and that at present all the organs of the body came under one medical fund, except the lungs, and extra was paid for these, although patients were treated at home. After further discussion, the matter was referred back for consideration.

### CITY OF CARDIFF HOSPITAL FOR THE FRENCH ARMY.

A movement for the provision of a further hospital for the treatment of French soldiers has been started at Cardiff. The French Government will supply the necessary buildings free of cost and will feed the patients. The amount required to equip and maintain this hospital of 120 beds for six months is roughly £2,500. The honorary services of two distinguished members of the medical profession have already been secured. The physician is Dr. Thomas Lewis, of Queen Anne Street, London, who is an M.D. Lond., F.R.C.P. Lond., and D.Sc. Wales. He was one of the ten who, out of seventy applicants, were awarded in 1910, under the Beit bequest, scholarships of the value of £250 per annum for three years for medical research. The surgeon is Mr. H. Morriston Davies, of Harley Street, who is an M.D., M.C. Camb., F.R.C.S. Eng. They will be assisted, it is understood, by doctors and nurses who will not receive any pay. We understand that the project has not received the financial support anticipated, but an effort is being made to bring home its claims to the generosity of the public.

### THE LOCAL GOVERNMENT BOARD AND SALARIES.

The Fulham Guardians, owing to the difficulty experienced in finding medical officers at their old scale of salaries, recently adopted a new scale. They suggested a salary of £160 per annum for the junior medical officer, £160 rising to £180 for the second assistant medical officer, and £200 rising to £225 for the assistant medical superintendent. The Local Government Board refused to sanction this scale in its entirety, but stated that they were prepared to sanction £140 rising to £160 for the junior medical officer, the salary suggested by the Guardians for the second assistant, and £200 without increment for the assistant medical superintendent. The Guardians have decided to point out to the Board that it is impossible to obtain good medical officers at these salaries, and to ask again for sanction for their original suggestion. It is strange that the Local Government Board should attempt to make these petty reductions, and it suggests that they are insufficiently informed as to the present difficulties in obtaining medical officers. If they persist in their objection it will only mean that *locums* will have to be obtained at a much increased cost. The days when medical officers could be obtained at the old inadequate salaries are gone for ever. It is now the duty of the Local Government Board to stimulate Guardians to offer salaries which will attract efficient candidates to the Poor-Law Service.

### LAYMEN AND THE HOSPITAL SPIRIT.

The note of joy, so pre-eminently a hospital note at Christmas, is, let us remember, no calculated thing, formally drawn up like a programme, or hung about the institution like a festoon; it is the leaven of the place, or, more correctly, it is the raising of the loaf consequent on the leaven being there. Joy is a quality of strength, and strength expresses itself in labour, and when you get the expression of pleasure in work you get, as William Morris told us, art. Well! the Christmas spirit in hospitals is a work of art, because it is the expression of pleasure in work—work for others dependent upon free and generous gifts of money, and personal service by nurses, doctors, committee-men, managers, visitors, ladies' leagues, and working-class collectors. What the spirit of personal service has done and is doing all hospital workers know: what it *is* in itself a Christmas afternoon spent in hospital will show you. Is not its service to the nation, this year especially, immense and freely given? Will you not, therefore, give of your best in time and money to support the voluntary hospitals, and make their habit of happiness and willingness to work your own in the New Year? Believe us, if the good work is to go on, your help is badly needed.

### A NAVAL SURGEON'S LIFE AT SEA.

One of the most interesting sidelights yet obtainable on the life of patrol ships at sea, and in particular on the duties of a naval surgeon on board a vessel so occupied, is contained in a letter published in the *Times* from Mr. Francis C. Searle, who was serving on H.M.S. *Good Hope* when she was lost in the action off Chile. From the letter, which was posted in a collier on October 29, we take the following brief extracts: "I'll start with the Spithead Review and Manœuvres, which lasted just over fourteen days. I was in the ship, as it was my war appointment. We paid off for four days, when I returned to the *Vernon*. Suddenly one night we got the order 'Mobilise,' and with an hour to pack two tin cases I found myself on board again here. Most of us brought only a change of wear, myself among them. Not even a watch overcoat, for the nights were warm. We were patrolling the Irish coast when war was declared, and when off the Southern Hebrides were ordered to sweep the Atlantic trade routes for hostile cruisers." He goes on to describe their voyage to Halifax, Bermuda, the West Indies and Trindad, the Falkland Islands, and remarks: "Five German cruisers against us. What's the betting on the field? Pray to your *penates* we may prevent them concentrating." Then in conclusion comes this picture of a surgeon's life aboard: "The days are full of monotony. First-aid lectures are boring to a degree, for we can't say too much, or it becomes a positive danger. Our casualties have so far been two. A stoker got buried in an avalanche of coal in a bunker, and a sergeant of Marines got an acute heart attack—one buried off Venezuela and the other off the Horn."

### FLORENCE NIGHTINGALE'S HOSPITAL LETTERS.

From the earliest times down to the battle of Waterloo is a wide field to cover even in relation to the comparatively restricted field of surgical instruments in war time, but this is the aim of the exhibition which was opened this week in the Wellcome Medical Museum, Wigmore Street. It may be said, perhaps, that the surgical knife, in all its modern protean forms, is a less alarming instrument to the layman's ignorance than were those in use in former days for amputation purposes. In reference to the remarkable article on bullet wounds which we publish on the next page, we may draw our readers' attention to the instruments for the extraction of bullets which are on view at the Wellcome Museum. They are apparently survivals from the Civil War of the days of Charles and Cromwell, when the process was first to enlarge the wound, and then to insert the extractor, which by means of a gimlet bored, or failed to bore, itself into the bullet. On this the *x*-rays is, indeed, a wonderful advance. Nelson's medicine chest, now doubly interesting from the fact of the war and also from the public's opportunity of being present at his death-bed, portrayed with such restraint and staged with such art in Mr. Thomas Hardy's "The Dynasts"; while the medicine chest belonging to Wellington, another protagonist of the same play, is also shown. Institutional readers, however, will note with peculiar interest the autograph letters from Florence Nightingale, the most interesting of which, from our point of view, is that written from the Barrack Hospital, Scutari, in 1855—nearly sixty years ago—in which her care for the last wishes of the soldiers is seen so practically. We may note this particularly, since our own reiteration of the paramount necessity for showing due respect to the dead in mortuaries and viewing-rooms is sometimes boggled at as supererogatory. But, indeed, the hospital must be a teaching centre in every locality on this as on other matters, and Florence Nightingale's respect for the wishes of the dying shows that that great administrator recognised to the full the supreme importance of insisting on the highest and most sympathetic standard on this point.

### THIS WEEK'S DRUG MARKET.

Business is less active, owing to the approach of Christmas and the annual stocktaking, but the tone of the market is healthy, and business will probably improve early in the New Year. Price fluctuations of importance have been few, and orders have, for the most part, been limited to what is necessary to satisfy immediate requirements. Opium and its alkaloids are unchanged, but the advanced prices are well maintained. Cod-liver oil is somewhat dearer, and if anything like a strong demand were to set in prices would most probably advance further. The advancing tendency of carbolic acid continues. Ipecacuanha is again dearer. Quinine is in very slow demand and prices are unchanged. Essence of lemon, oil of orange, and essence of bergamot have a slightly lower tendency in price. Sodium salicylate and salicylic acid are in diminished supply, and prices have an upward tendency. Potassium permanganate has declined in value. Cream of tartar tends downwards in price.

